# Prognostic value of the model for end-stage liver disease excluding INR score (MELD-XI) in patients with adult congenital heart disease

**DOI:** 10.1371/journal.pone.0225403

**Published:** 2019-11-19

**Authors:** Ryo Konno, Shunsuke Tatebe, Koichiro Sugimura, Kimio Satoh, Tatsuo Aoki, Masanobu Miura, Hideaki Suzuki, Saori Yamamoto, Haruka Sato, Yosuke Terui, Satoshi Miyata, Osamu Adachi, Masato Kimura, Yoshikatsu Saiki, Hiroaki Shimokawa

**Affiliations:** 1 Department of Cardiovascular Medicine, Tohoku University Graduate School of Medicine, Sendai, Japan; 2 Department of Evidence-Based Cardiovascular Medicine, Tohoku University Graduate School of Medicine, Sendai, Japan; 3 Department of Cardiovascular Surgery, Tohoku University Graduate School of Medicine, Sendai, Japan; 4 Department of Pediatrics, Tohoku University Graduate School of Medicine, Sendai, Japan; Policlinico Universitario Agostino Gemelli IRCCS, ITALY

## Abstract

Patients with adult congenital heart disease (ACHD) are at increased risk of developing late cardiovascular complication. However, little is known about the predictive factors for long-term outcome. The Model for End-Stage Liver Disease eXcluding INR (MELD-XI) score was originally developed to assess cirrhotic patients and has the prognostic value for heart failure (HF) patients. In the present study, we examined whether the score also has the prognostic value in this population. We retrospectively examined 637 ACHD patients (mean age 31.0 years) who visited our Tohoku University hospital from 1995 to 2015. MELD-XI score was calculated as follows; 11.76 x ln(serum creatinine) + 5.11 x ln(serum total bilirubin) + 9.44. We compared the long-term outcomes between the high (≥10.4) and the low (<10.4) score groups. The cutoff value of MELD-XI score was determined based on the survival classification and regression tree (CART) analysis. The major adverse cardiac event (MACE) was defined as a composite of cardiac death, HF hospitalization, and lethal ventricular arrhythmias. During a mean follow-up period of 8.6 years (interquartile range 4.4–11.4 years), MACE was noted in 51 patients, including HF hospitalization in 37, cardiac death in 8, and lethal ventricular arrhythmias in 6. In Kaplan-Meier analysis, the high score group had significantly worse MACE-free survival compared with the low score group (log-rank, P<0.001). Multivariable Cox regression analysis showed that the MELD-XI score remained a significant predictor of MACE (hazard ratio 1.36, confidence interval 1.17–1.58, P<0.001) even after adjusting for patient characteristics, such as sex, functional status, estimated glomerular filtration rate, and cardiac function. Furthermore, CART analysis revealed that the MELD-XI score was the most important variable for predicting MACE. These results demonstrate that the MELD-XI score can effectively predict MACE in ACHD patients, indicating that ACHD patients with high MELD-XI score need to be closely followed.

## Introduction

Along with the advances in the treatment of congenital heart disease (CHD), more than 90% of CHD patients are expected to reach adulthood [1]. As a result, the number of patients with adult congenital heart disease (ACHD) has been progressively increasing worldwide [2]. Accumulating epidemiological evidence showed that these patients are not cured and at high risk of developing late cardiovascular complications, such as heart failure (HF), lethal arrhythmias, pulmonary hypertension, and even sudden cardiac death [3–6]. We have recently demonstrated that HCV antibody positivity predicts cardiovascular outcomes in selected ACHD patients [7]. However, little evidence is available regarding the risk factors that affect the long-term prognosis in ACHD patients in general.

The Model for End-Stage Liver Disease (MELD) score was originally developed to assess the short-term survival of patients with liver cirrhosis undergoing transjugular intrahepatic portosystemic shunt (TIPS) procedures [8]. This score uses 3 objective biochemical values, including serum total bilirubin, creatinine, and international normalized ratio for prothrombin time (INR), and effectively reflects hepatic and renal dysfunction after TIPS procedures [8]. Since congestion due to impaired cardiac function causes multiple end organ dysfunctions including liver and kidneys, the MELD score has been reported to be an excellent predictor for major cardiac adverse events (MACE) in HF patients [9]. Furthermore, the Model for End-Stage Liver Disease eXcluding INR (MELD-XI) score was subsequently developed as an alternative to the original MELD score to assess cirrhotic patients receiving anticoagulation therapy with vitamin K antagonists [10]. The MELD-XI score has also been reported to predict poor outcomes in HF patients [11–13].

In the present study, we thus examined whether the MELD-XI score could also be a useful predictor for long-term outcomes in ACHD patients, by conducting a retrospective cohort study with our AHCD database in the Tohoku University Hospital.

## Materials and methods

### Study population

A total of 1,031 ACHD patients older than 18 years visited our hospital from 1995 to 2015. Patients with follow-up time of less than one year (n = 317), or limited data unable to calculate the MELD-XI score (n = 76) were excluded. We also excluded a patient with dialysis-dependent renal failure. Finally, 637 ACHD patients (47% male, mean age 31.0±14.5 years) were enrolled in the present study. The study protocol was in accordance with the ethical guidelines of the 1975 Declaration of Helsinki and was approved by the Ethics Committee of the Tohoku University Graduate School of Medicine (No. 2016-1-510, UMIN000025734). Informed consent was obtained by the opt-out method.

### Data collection

In order to calculate the first MELD-XI score, we collected the baseline data from medical records regarding age, sex, diagnosis of CHD, New York Heart Association (NYHA) functional class, cardiac function, renal function, liver function, anemia, and other clinical characteristics. Anemia was defined as hemoglobin level <13.0 g/dL for men and <12.0 g/dL for women. Liver cirrhosis was defined based on clinical, laboratory, and imaging findings. Estimated glomerular filtration rate (eGFR) was calculated by using Modification of Diet in Renal Disease formula modified for Japanese [14]. MELD-XI score was calculated as previously reported [10]; MELD-XI = 5.11× ln (serum total bilirubin in mg/dL) + 11.76 × ln (serum creatinine in mg/dL) + 9.44, where ln means the natural logarithm. Any values less than 1 were given the lower limit value of 1 to prevent a negative score. Thus, minimum possible MELD-XI score was 9.44. Systemic ventricular ejection fraction (SVEF) and subpulmonary ventricular (SPV) function were assessed by echocardiography or cardiac magnetic resonance imaging. SPV dysfunction was defined as SPV ejection fraction ≤45% or SPV fractional area change ≤35%. Valvular disease was defined as moderate or severe valvular stenosis or regurgitation on echocardiography. Pulmonary hypertension was defined as mean pulmonary artery pressure ≥25mmHg on right heart catheterization or estimated pulmonary artery systolic pressure ≥40mmHg on echocardiography. The complexity of CHD was assigned according to the Bethesda classification [15].

### Definition of the MACE

The MACE included cardiac death, HF hospitalization, and lethal ventricular arrhythmias. Lethal ventricular arrhythmias were defined as composite arrhythmic events, including ventricular fibrillation and sustained ventricular tachycardia. Hospitalization for HF was defined as an unplanned hospitalization due to worsening HF signs and symptoms of NYHA class III/IV. We retrospectively observed the patients until the occurrence of MACE or the end of August 2018.

### Statistical analysis

Continuous variables are expressed as the mean ± standard deviation (SD) and were assessed by the Student t-test or Wilcoxon rank-sum test as appropriate. Categorical variables are expressed as proportions and were analyzed by Fisher exact test. Patients were divided into 2 groups according to their MELD-XI score; the low score (MELD-XI <10.4, n = 532) and the high score (MELD-XI ≥10.4, n = 105) groups. The cutoff value of MELD-XI score was determined based on the survival classification and regression tree (CART) analysis [16]. MACE-free survival curves were estimated by the Kaplan-Meier method and compared between the high and the low score groups by using the log-rank test. Univariable and multivariable Cox proportional hazards regression analyses were performed to evaluate the association of baseline variables with the MACE. In the multivariable models, we selected the variables by using a backward stepwise method with variables entered if P-value was less than 0.1 in univariable analysis. Event rate of the MACE and its each component were compared between the high and the low score groups using the Wald method. Survival CART analysis was performed to develop a prediction model for identifying high-risk patients for MACE using variables with P-value less than 0.1 in univariable Cox regression analysis. Survival CART analysis was used not only to determine the risk factors for MACE but to identify the optimal cut-off points and the relative importance of various risk factors. We performed a logistic regression analysis to determine the association of SVEF <50% with renal and hepatic function. Results were considered to be statistically significant at P<0.05. All statistical analyses were performed using JMP® Pro 14.1 (SAS Institute Inc., Cary, NC, USA) and R version 3.5.1 (The R Project for Statistical Computing; https://www.r-project.org/).

## Results

### Baseline characteristics of the study population

Of the 637 ACHD patients included in the present study, the most common diagnosis of CHD was ventricular septal defect (23%), followed by tetralogy of Fallot (19%), and atrial septal defect (12%) (Table 1). There were 32 (5.0%) patients with Fontan circulation and 20 (3.1%) with Eisenmenger syndrome. Baseline characteristics of the patients are shown in Table 2. The mean MELD-XI score for all patients was 9.94±1.14 (interquartile range [IQR] 9.44–9.93). The high score group consisted of 105 patients and the low score group 532 patients (Table 2). The prevalence of eGFR <90 ml/min/1.73m^2^ and total bilirubin >1.0 mg/dl in the entire population was 31% and 25%, respectively. Hepatitis virus infection was detected in 71, including positive hepatitis B virus antigen in 6 and positive HCV antibody in 65. The prevalence of hepatitis virus infection was comparable between the 2 groups (Table 2). Moreover, MELD-XI score was comparable between the patients with and those without hepatitis virus infection (10.07±1.13 vs. 9.97±1.23, P = 0.249). Compared with the low score group, the high score group was characterized by a male predominance, increased γ-glutamyl transpeptidase (GGT) and aspartate aminotransferase (AST), a lower platelet count, higher prevalence of great complexity of CHD, NYHA functional class ≥II, Fontan circulation, renal insufficiency (eGFR <60 ml/min/1.73m^2^), SVEF<50%, and SPV dysfunction (Table 2).

**Table 1:** Diagnoses of congenital heart disease.

Diagnosis	n (%)
VSD	144 (23)
TOF	118 (19)
ASD	77 (12)
Marfan syndrome	47 (7.4)
Congenital valvular disease	37 (5.8)
AVSD	31 (4.9)
Complete TGA	29 (4.6)
Pulmonary atresia	26 (4.1)
DORV	21 (3.3)
ccTGA	19 (3.0)
Aortic coarctation	17 (2.7)
TAPVR/PAPVR	16 (2.5)
Single ventricle	13 (2.0)
Ebstein disease	11 (1.7)
PDA	10 (1.6)
Tricuspid atresia	8 (1.3)
Others	13 (2.0)
All patients	637 (100)

ASD, atrial septal defect; AVSD, atrioventricular septal defect; ccTGA, congenitally corrected transposition of the great arteries; DORV, double outlet right ventricle; PAPVR, partial anomalous pulmonary venous return; PDA, patent ductus arteriosus; TAPVR, total anomalous pulmonary venous return; TGA, transposition of the great arteries; TOF, Tetralogy of Fallot; VSD, ventricular septal defect.

**Table 2:** Baseline characteristics of all patients and by MELD-XI score status.

Variables	All (n = 637)	MELD-XI score		P value
		High (≥10.4) (n = 105)	Low (<10.4) (n = 532)	
Age (years), mean	31.0 ± 14.5	29.1 ± 13.0	31.4 ± 14.8	0.084
Male sex	298 (47)	75 (71)	223 (42)	<0.001
CHD complexity				<0.001
Simple	254 (40)	25 (24)	229 (43)	
Moderate	242 (38)	41 (39)	201 (38)	
Great	141 (22)	39 (37)	102 (19)	
Repaired status	498 (78)	88 (84)	410 (77)	0.155
NYHA functional class ≥II	194 (30)	51 (49)	143 (27)	<0.001
Systemic right ventricle	32 (5.0)	6 (5.7)	26 (4.9)	0.633
Fontan circulation	32 (5.0)	14 (13)	18 (3.4)	<0.001
Eisenmenger syndrome	20 (3.1)	4 (3.8)	16 (3.0)	0.758
Tetralogy of Fallot	118 (19)	18 (17)	100 (19)	0.784
PMI in childhood	18 (2.8)	1 (1.0)	17 (3.2)	0.334
Hypertension	39 (6.1)	4 (3.8)	35 (6.6)	0.374
Dyslipidemia	20 (3.1)	1 (1.0)	5 (3.6)	0.225
Diabetes mellitus	18 (2.8)	3 (2.9)	15 (2.8)	1.0
Liver cirrhosis	5 (0.8)	2 (1.9)	3 (0.6)	0.192
Hepatitis virus infection	71/519 (14)	18/93 (19)	53/426 (12)	0.095
HBV antigen positivity	6/519 (1.2)	1/93 (1.1)	5/426 (1.2)	
HCV antibody positivity	65/519 (13)	17/93 (18)	48/426 (11)	
Serum creatinine >1.0 mg/dl	9/637 (1.4)	8 (7.6)	1 (0.2)	<0.001
eGFR (ml/min/1.73m^2^)				0.021
≥90 ml/min/1.73m^2^	439 (69)	69 (66)	370 (70)	
60 to 89 ml/min/1.73m^2^	175 (27)	27 (26)	148 (28)	
<60 ml/min/1.73m^2^	23 (3.6)	9 (8.6)	14 (2.6)	
mean	105.2 ± 30.6	101.6 ± 33.8	106.0 ± 29.9	0.264
Total bilirubin >1.0 mg/dl	161/637 (25)	98 (93)	63 (12)	<0.001
GGT >50 mg/dl	122/629 (19)	31/105 (30)	91/524 (17)	0.007
AST >40 IU/dl	38/637 (6.0)	11/105 (10)	27/532 (5.1)	0.042
ALT >40 IU/dl	60/637 (9.4)	13/105 (12)	47/532 (8.8)	0.272
Anemia	104/637 (16)	6/105 (5.7)	98/532 (18)	<0.001
Serum albumin <3.5 g/dl	29/613 (4.7)	3/103 (2.9)	26/510 (5.1)	0.451
Platelet count <150,000 /μl	98/637 (15)	25/105 (24)	73/532 (14)	0.012
MELD-XI score	9.94 ± 1.14	12.05 ± 1.49	9.53 ± 0.25	<0.001
SVEF (%), mean	66.2 ± 11.7	64.9 ± 13.2	66.4 ± 11.4	0.314
<50%	43/602 (7.1)	13/98 (13)	30/504 (6.0)	0.017
SPV dysfunction	54/542 (10)	15/79 (19)	39/463 (8.4)	0.007
Valvular disease	168/597 (28)	30/96 (31)	138/501 (28)	0.459
Pulmonary hypertension	80/595 (13)	16/96 (17)	64/499 (13)	0.327

Continuous variables and categorical variables are expressed as mean ± SD and number (percentage), respectively. ALT, alanine aminotransferase; AST, aspartate aminotransferase; CHD, congenital heart disease; eGFR, estimated glomerular filtration rate; GGT, γ-glutamyl transpeptidase; HBV, hepatitis B virus; HCV, hepatitis C virus; MELD-XI, model for end-stage liver disease excluding international normalized ratio; NYHA, New York Heart Association; PMI, pacemaker implantation; SD, standard deviation; SPV subpulmonary ventricle; SVEF, systemic ventricular ejection fraction.

### Incidence rates of MACE

**Fig 1:**
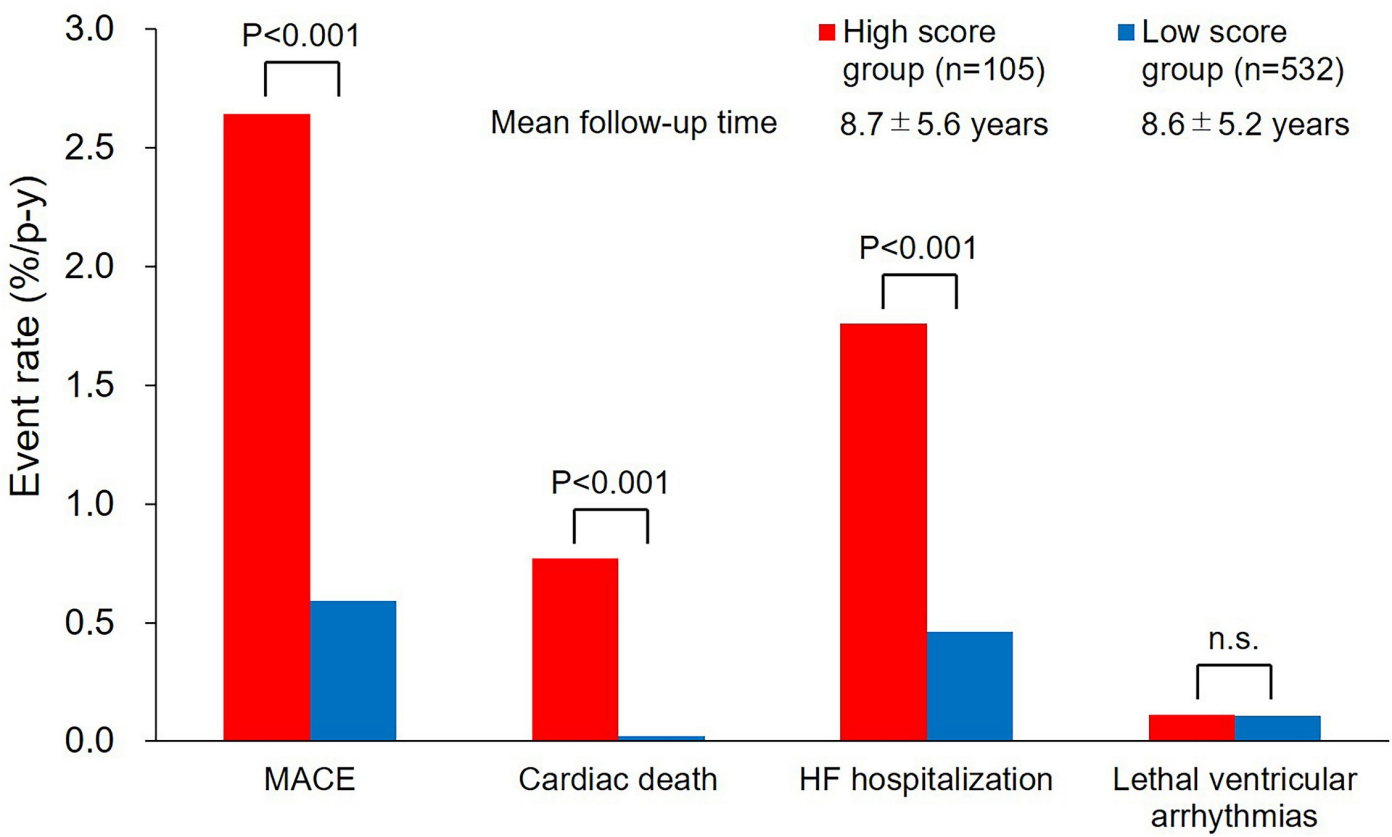
Comparison of event rate between the high and the low MELD-XI score groups. During the mean follow-up of 8.6±5.3 years, MACE was noted in 51 patients. The incidence rate of MACE, cardiac death, and HF hospitalization was significantly higher in the high score group compared with the low score group. In contrast, the incidence rate of lethal ventricular arrhythmias was comparable between the two groups. HF, heart failure; MACE, major adverse cardiac events; MELD-XI, model for end-stage liver disease excluding international normalized ratio; p-y, person-year.

During the mean follow-up period of 8.6±5.3 years (IQR 4.4–11.4 years), MACE was noted in 51 (8.0%) patients, including HF hospitalization in 37, cardiac death in 8, and lethal ventricular arrhythmias in 6. The incidence rate of MACE was 0.93% per person-year in overall patients. The incidence rate of MACE for 3 CHD complexity groups (% per person-year) was 0.5%, 1.1%, and 1.4% for the simple, moderate, and severe groups, respectively (P = 0.037). MACE occurred more frequently in the high score group compared with the low score group (2.6 vs. 0.6% per person-year, P<0.001) (Fig 1). Moreover, the incidence rate of cardiac death and HF hospitalization was significantly higher in the high score group compared with the low score group (0.8 vs. 0.02% per person-year, P<0.001, 1.8 vs. 0.5% per person-year, P<0.001, respectively) (Fig 1). In contrast, there was no significant difference in the incidence rate of lethal ventricular arrhythmias (0.11 vs. 0.11% per person-year, P = 0.996). In the subgroup of 32 patients with Fontan circulation, only 2 patients developed MACE for HF hospitalization.

### Survival analysis

**Fig 2:**
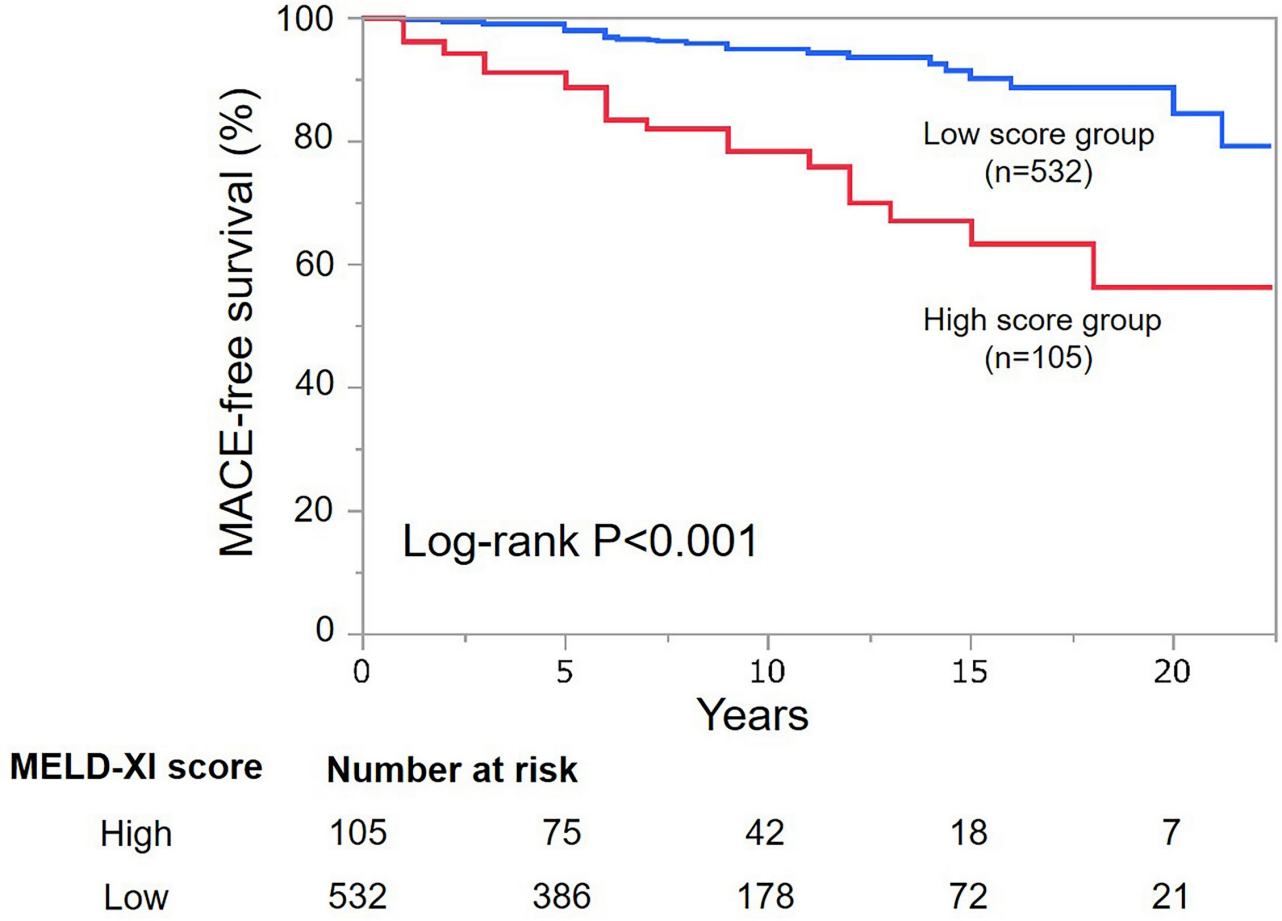
Kaplan-Meier curves according to the MELD-XI score. The high score group had significantly poor MACE-free survival compared with the low score group (log-rank, P<0.001). MACE, major adverse cardiac events; MELD-XI, model for end-stage liver disease excluding international normalized ratio.

**Fig 3:**
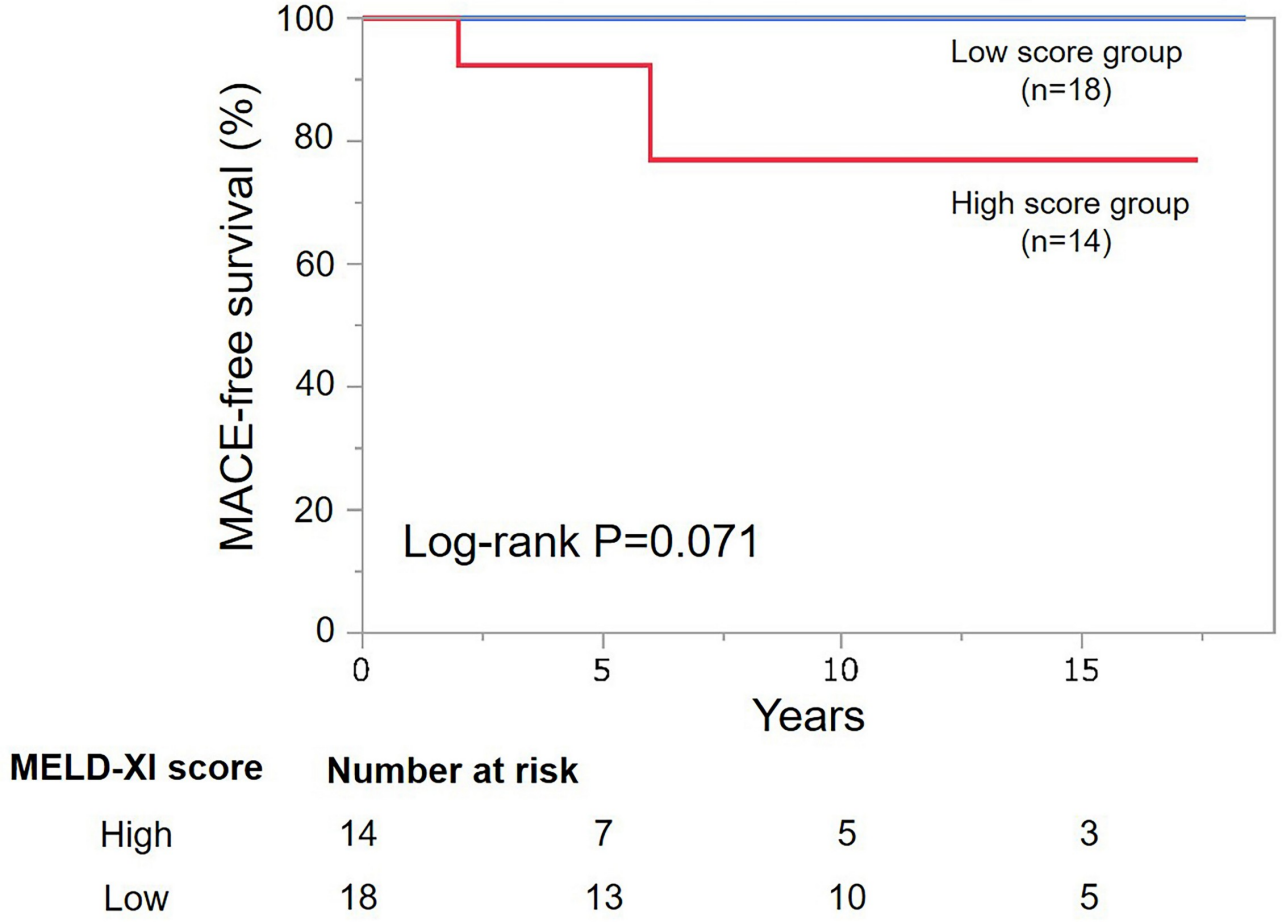
Kaplan-Meier curves for a subgroup of patients with Fontan circulation. MACE-free survival was not significantly different between the high and the low score groups (log-rank, P = 0.071). MACE, major adverse cardiac events; MELD-XI, model for end-stage liver disease excluding international normalized ratio.

Kaplan-Meier survival analysis for MACE is shown in Figs 2 and 3. The overall estimated 5-years MACE-free survival rate for all ACHD patients was 96% (95% CI: 94–98%). MACE-free survival was significantly lower in the high score group compared with the low score group (log-rank, P<0.001). The estimated 5 and 10-years MACE-free survival rates were 89 and 78% for the high score group, and 98% and 95% for the low score group, respectively (Fig 2). In patients with Fontan circulation, there was no significant difference in MACE-free survival between the high and the low score groups (log-rank, P = 0.071) (Fig 3).

**Fig 4:**
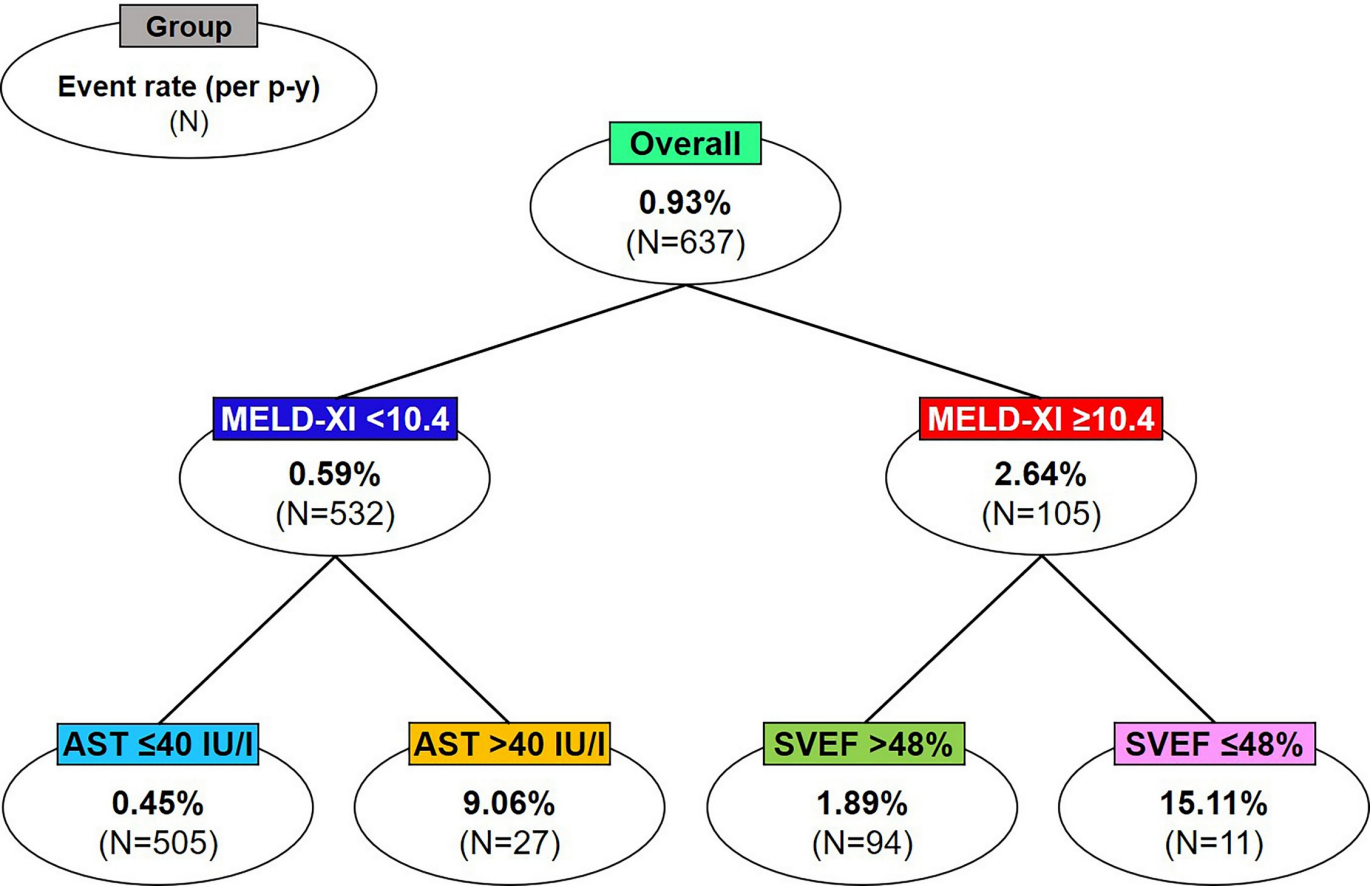
Survival CART analysis and prognostic value of the MELD-XI score in ACHD patients. The survival CART analysis revealed that MELD-XI score was the primary discriminator of MACE (P<0.001), indicating that the score is the most useful prognostic factor for MACE in ACHD patients. ACHD, adult congenital heart disease; AST, aspartate aminotransferase; CART, classification and regression tree; MACE, major adverse cardiac events; MELD-XI, model for end-stage liver disease excluding international normalized ratio; p-y, person-year; SVEF, systemic ventricular ejection fraction.

Univariable Cox proportional hazard analysis showed that 17 risk factors were significantly associated with MACE, including MELD-XI score, age, male sex, great complexity of CHD, NYHA functional class ≥II,HCV antibody positivity, serum creatinine, eGFR, total bilirubin, GGT, AST, alanine aminotransferase (ALT), platelet count, SVEF<50%, SPV dysfunction, valvular disease, and pulmonary hypertension (Table 3). Multivariable Cox analysis revealed that MELD-XI score remained an independent predictor of MACE (HR 1.36, 95% CI: 1.17–1.58, P<0.001) even after adjustment of sex, NYHA functional class, eGFR, ALT, SVEF<50%, and the presence of valvular disease and pulmonary hypertension (Table 4). In survival CART analysis, MELD-XI score was identified as a primary discriminator of MACE (P<0.001), indicating that the score was the most useful variable in the risk prediction model in terms of explanatory power (Fig 4). The subgroup of 11 patients with the highest MACE rate was characterized by 2 criteria; MELD-XI ≥10.4 and SVEF ≤48% (Fig 4). This group had a 16-fold increased risk of MACE compared with the entire group (15.1 vs. 0.93% per person-year, P<0.001). In the logistic regression analysis, SVEF <50% was associated with total bilirubin >1.0 mg/dl (P = 0.010) and GGT >50 mg/dl (P = 0.004) but not with AST >40 IU/l (P = 0.376), ALT >40 IU/l (P = 0.301), or eGFR (P = 0.408).

**Table 3:** Univariable Cox regression analysis for prediction of MACE.

Variables	HR (95% CI)	P value
Age (years)	1.02 (1.00–1.04)	0.028
Male sex	2.64 (1.49–4.90)	<0.001
CHD complexity		
Simple	Ref	
Moderate	2.00 (0.98–4.41)	0.057
Great	2.69 (1.25–6.10)	0.011
Repaired status	1.65 (0.76–4.34)	0.223
NYHA functional class ≥II	3.31 (1.87–6.10)	<0.001
Systemic right ventricle	1.01 (0.24–2.76)	0.986
Fontan circulation	0.83 (0.13–2.67)	0.784
Eisenmenger syndrome	3.02 (0.91–7.48)	0.068
Tetralogy of Fallot	1.57 (0.84–2.81)	0.139
PMI in childhood	1.14 (0.19–3.70)	0.856
Hepatitis virus infection	1.83 (0.93–3.36)	0.078
HBV antigen positivity		0.281
HCV antibody positivity	1.99 (1.02–3.67)	0.045
Hypertension	0.85 (0.14–2.74)	0.811
Dyslipidemia	1.20 (0.21–4.22)	0.726
Diabetes mellitus	0.70 (0.04–3.19)	0.706
Liver cirrhosis	4.00 (0.65–13.0)	0.114
Serum creatinine >1.0 mg/dl	7.13 (1.72–19.7)	0.011
eGFR (ml/min/1.73m^2^)	0.98 (0.97–0.99)	0.004
Total bilirubin >1.0 mg/dl	2.90 (1.67–5.05)	<0.001
GGT >50 mg/dl	2.76 (1.54–4.81)	<0.001
AST >40 IU/l	3.11 (1.51–5.87)	0.003
ALT >40 IU/l	2.35 (1.17–4.36)	0.018
Anemia	1.29 (0.59–2.53)	0.506
Serum albumin <3.5 g/dl	1.29 (0.31–3.52)	0.678
Platelet count <150,000 /μl	2.75 (1.48–4.89)	0.002
MELD-XI score	1.46 (1.26–1.65)	<0.001
SVEF (%)	0.95 (0.93–0.97)	<0.001
SVEF <50%	7.83 (3.93–14.7)	<0.001
SPV dysfunction	4.92 (2.47–9.26)	<0.001
Valvular disease	2.87 (1.63–5.06)	<0.001
Pulmonary hypertension	2.81 (1.49–5.08)	0.002

CI, confidence interval; HR, hazard ratio; MACE, major adverse cardiac event; Ref, reference. See Table 2 for other abbreviations.

**Table 4:** Multivariable Cox regression analysis for prediction of MACE.

Variables	HR (95% CI)	P value
Male sex	1.66 (0.85–3.26)	0.140
NYHA functional class ≥II	2.05 (1.07–3.92)	0.030
eGFR (ml/min/1.73m^2^)	0.99 (0.98–1.00)	0.007
ALT >40 IU/l	2.31 (1.14–4.67)	0.020
MELD-XI score	1.36 (1.17–1.58)	<0.001
SVEF <50%	3.78 (1.84–7.79)	<0.001
Valvular disease	1.91 (1.03–3.56)	0.042
Pulmonary hypertension	1.84 (0.90–3.76)	0.095

Association of MELD-XI score with the composite endpoint, adjusted for sex, CHD severity, New York Heart Association functional class, increased AST, reduced SVEF, valvular disease, and pulmonary hypertension (model P<0.001). CI, confidence interval; HR, hazard ratio; MACE, major adverse cardiac event; Ref, reference. See Tables 2 and 3 for other abbreviations.

## Discussion

In the present study, we demonstrated that ACHD patients at increased risk of MACE were characterized by advanced NYHA functional class, decreased eGFR, increased ALT, elevated MELD-XI score, decreased SVEF, and the presence of valvular disease. Importantly, the MELD-XI score was the most useful variable among these risk factors. To the best of our knowledge, this is the first report that identifies the MELD-XI score as a novel predictor of long-term prognosis of ACHD patients.

Liver and renal dysfunctions are common in HF patients and associated with poor prognosis [17]. These interactions are known as cardio-renal and cardio-hepatic syndromes [17]. Both increased serum total bilirubin and decreased eGFR have been reported as risk factors for adverse cardiovascular outcomes not only in HF patients [18, 19] but also in ACHD patients [20, 21]. Consistent with these reports, we noted a high prevalence of increased total bilirubin (25%) and reduced eGFR (31%) in our ACHD patients, both of which were significantly associated with MACE. Although the exact mechanism for this association remains to be elucidated, venous congestion and insufficient organ perfusion are thought to be hemodynamically involved in the development of organ-related co-morbidities in HF patients [17].

The MELD score was initially developed to predict early mortality in patients undergoing TIPS procedures [8]. Since the MELD score is composed of 3 laboratory values reflecting hepatic and renal dysfunctions, it has emerged as a novel biomarker to assess multi-organ dysfunctions in HF. Increased MELD score has been reported to predict poor cardiovascular outcome in patients with advanced HF referred for ventricular assist device implantation [22], or heart transplantation [23, 24]. However, there could be an overestimation in the risk prediction when MELD score is applied to patients with warfarin therapy. In this situation, INR is artificially elevated by antagonizing vitamin K-dependent pathways, leading to a high MELD score. In fact, anticoagulation with warfarin is commonly used in patients with various cardiovascular conditions, such as HF, atrial fibrillation, venous thromboembolism, mechanical heart valve, and ACHD including Fontan circulation [25, 26]. Thus, the MELD-XI score, a modification of the MELD score, was subsequently developed for better risk prediction in cirrhotic patients with warfarin therapy [10]. The MELD-XI score omits INR from the equation and has been validated to predict the prognosis in HF patients effectively [11–13]. Three groups have previously reported the usefulness of the MELD-XI score in the field of ACHD. Assenza et al. reported that a higher MELD-XI score was associated with worse survival for the composite endpoint of death or cardiac transplantation in Fontan patients [27]. Lewis et al. and Adams et al. independently reported that the MELD-XI score predicted mortality and morbidity in ACHD patients undergoing heart transplantation [28, 29]. In the present study, we were able to demonstrate that high MELD score has an important prognostic value for MACE in the entire ACHD patients. Thus, the MELD-XI score could be useful as a novel and useful predictor of future cardiovascular events in patients with a wide spectrum of both acquired and congenital heart disease, regardless of warfarin use.

In the present study, contrary to the previous report [27], MACE-free survival was comparable between the high and low MELD-XI score groups in Fontan patients. This discordance may be attributable to the smaller number of Fontan patients and the lower incidence of MACE event in our study compared with the previous report (32 vs. 96 patients, 0.76 vs. 3.0% per person-year, respectively) [27]. However, the MACE rate in the present study was comparable with other reports [30, 31].

Survival CART analysis revealed that the MELD-XI score was the primary discriminator of MACE. Assenza et al. reported that MELD-XI score was a better predictor of adverse events than serum creatinine in patients with Fontan circulation [27]. We consider that high MELD-XI score comprehensively represents the severity of multi-organ dysfunctions in HF associated with ACHD, as the elevation results from hemodynamic effects of venous congestion and low cardiac output, thus predicting future cardiovascular events better than a single measurement of total bilirubin or creatinine. Indeed, in the present study, SVEF was also associated with MACE and was identified as a primary discriminator in the subgroup of high MELD-XI group. In the logistic regression analysis, SVEF <50% was associated with increased total bilirubin and GGT but not with eGFR. Contrary to our study, it was previously reported that renal dysfunction was associated with systemic ventricular dysfunction [21]. This discordance may be attributable to younger age and relatively lower prevalence of reduced eGFR in our study compared with the previous report (31.0 ± 14.5 vs. 36.0 ± 14.2 years, 31% vs. 50%, respectively) [21].

### Limitations

Several limitations should be mentioned for the present study. First, the present study was a single-center retrospective study and included selected ACHD patients who visited our institute from 1995 to 2015. In addition, the cohort consisted of patients with a wide variety of CHD, including Fontan circulation, systemic right ventricle, and Eisenmenger syndrome. Thus, the present results remain to be confirmed in future multi-center studies. Second, there were missing data due to the retrospective nature of the present study. The variable with the highest percentage of missing data was HCV antibody status (19%), followed by SPV dysfunction (15%). These two variables were excluded from the multivariable analysis. Third, we could not obtain either detailed patient history, such as previous hospital admission, or pathophysiology, such as extracardiac shunt and hemodynamic data from cardiac catheterization. Further studies are needed to clarify whether these factors could be independent predictors of MACE in ACHD patients. Forth, the two MELD-XI groups had different characteristics, in terms of CHD complexity and the prevalence of SPV dysfunction, SVEF <50%, and Fontan circulation. This may affect the outcome of the present study. Finally, we were unable to assess the association between the MELD-XI score and central venous pressure or cardiac output because of the lack of hemodynamic data. Further studies are needed to evaluate whether early intervention for decreasing the MELD-XI score could improve the clinical outcome of ACHD patients.

## Conclusions

In the present study, we were able to demonstrate that a higher MEDL-XI score was significantly associated with MACE in ACHD patients. Thus, the MELD-XI score is useful in identifying ACHD patients at high risk for developing late cardiovascular events. ACHD patients with high MELD-XI score need to be closely followed.

## Supporting information

S1 Dataset The data collected from medical records. (XLSX)
